# Supplementary material for: Method of Monitoring 26S Proteasome in Cells Revealed the Crucial Role of PSMA3 C-Terminus in 26S Integrity
Source: Biomolecules. 2023 Jun 15;13(6):992. doi: 10.3390/biom13060992 (PMC10296302; doi:10.3390/biom13060992)
Supplement: Supplementary file 1 [file biomolecules-13-00992-s001.zip › biomolecules-2382532-supplementary.pdf]

Supporting Information

Method of monitoring 26S proteasome in cells revealed the crucial role of PSMA3 C-terminus in 26S integrity

Shirel Steinberger, Julia Adler, and Yosef Shaul

TACCTTTCAGGTAGTAGTTGAATTCAGGTAGTATTTGTTCAGTTTTTTTTTTTCCCTTCATGT
TCTAAGACCAGCTTGAGAGGCAAAGGTTGTACCACTGAGCTCTAGTTGTTGTTACCTAAAAA
GGCCTTGTTTTAAATTTCTGTGATACCTAAGAATTTCAAATCTGGGTTGTCATGGATTCTTTA
TTCTTTTTTTCTCCCTTAAAAAGTTACATTTTAGATGAAATCCCCTTTTTTAAAATGGGCAAA
GCAATAATTCTACATCATTTCTCCCCTTCCCTTCCACTTGTTTAGACTAAGATATGTTAGAGA
GGGAAAGGGTCGTTGTTTTAGTAAATACTATTGCTGTTGACATGTTAATACTATTGCTGTTGA
CATGTTTACTGATGGGCTGTGTTCCATAATTTTGTTTTAGGTCTTTTGTTTGAAACAGTTTACT
GTTTTTATCAGTTTTGGTCCCTAATTTTTCCTAACCTACAGTTTTTCTCTGAGTACATATGGTT
TCATTGTTTGATCTACTTTCTATCTATCTGAATATGAACTTCTAGGATCATGTTTATTCTAGTA
GATGATGACTTAAAGCCTGCAGTATAGGAGGGACAACGTCAACTACTGCATGTGCAATAAC
AAGCTTGAAGGGAAGCTAAATGTTTGTTACAAATTTAAGACAGTATTTTAATGCCGTTTGCA
TTTTTCTAAGAATTTTCTATAAAGCTAATTCTGTTATTTTTTGTCTCTAAATTAGGGAACTGTC
CAGGTTTATTGCTGCCGGGAGACTACACTGCAAAATAGATAAAGTGAATGAAATAGTAGAA
ACCAACAGGTACTCTCATTTCTCAGAATAAGGGGCATTCCTAAATTTTAAAAGTAGGTCAAC
TATTGTCATGGAATAATGTGACTGGTAAATAATTCATTTTTTCTTGAATTTATTTATAGACCT
GATAGCAAGAACTGGCAGTACCAAGAAACTATCAAGAAAGGAGATCTGCTACTAAACAGAG
TTCAAAAACTTTCCAGAGTAATTAATATG.

The sequence of the donor 964 bp is:

TAAAGCCATGTAACTAACAAATGATTTGCTTTAGAGATAATTATTTGGAATTTTTATAGCTTA
CTTCACAATGTGCCCAGGTCAGCTGTATAAAATAAATACTGCATTGTTGTTTCTTTCCATTTA
TTTTTTTCTTAAACACATAGGCCTTCTAGAATCGGGTCCACTTATGTTAAAATAATGTCACTT
GAATTATTCATTAATGAAAATCTGGTTTATAGGGTAAGGTTGCAGCTGGGTTCAGCTAGGTT
CAGCTCCTGACTGCCTCATCAGTAAAATACGTCAGTAAAATACGGATGTTGACACAGCCCAT
TATCGCTTACCTTTAAATCTCTTACCTGTAAGCTTCCCGATTTTAGAAAGAAGAGTAGTGGCC
TAAAATTAAACAAACAGTGTCTCCCACTTTTAGTATACAAAAGATAGAAGATAAAGAAAAT
GTGTAATAAGCTTTCCCTCAGAAACAAGTAACCATATAAGGCAGGAAGTTACTCTTTTTTAG
GAAGCTGACATAACCTGGATTACAGAATCTACCAAGGCTGATCTCAGTTTTCTAAAACCTTA
TATGTAAGTATTTATTAGCAAACTGAATCTTTTTTCCAAAAGTAGGACCATAGTAATAATAG
CAGTGAGGAAAACAAAGTTGTTCCTCTGTTTGTTTGAAACAGTTTATTAGTTTTATCAGTTTT
ATTCAGTGTGTCAGATAGGAATAAGTGCTATAAGAAGTATAAAACCAGATAGACAGACAGA
TAGATAGATAGATAGATAGAGATACAGGGTATAGGCCGTCTTCTGAGGTGTTGTGGAAGGC
TTCTATGGTAAGGTGACATTTAAGTGAAAAGGAGGGAGAGCCCATGAACGTATGGAGGGAA
GAAAGACGAGGAAAGTCACTGGCAATTTTAAGTGCGGATTTGGCTTACCTTTTACGTCTCAC
TTACGGCATTCGGTGGCATGGATTGTACCGCGG.

Figure S1: The sequence of the donor DNA in CRISPR-Cas9 editing of PSMD6.

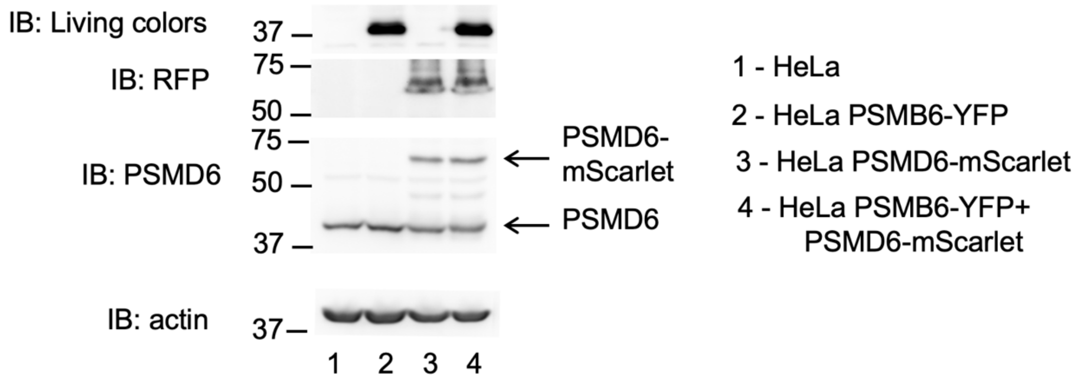

Figure S2: Evidence for heterozygosity of the PSMD6-mScarlet cells. Extracts from the indicated cells were prepared and analyzed by western blotting using the indicated antibodies.

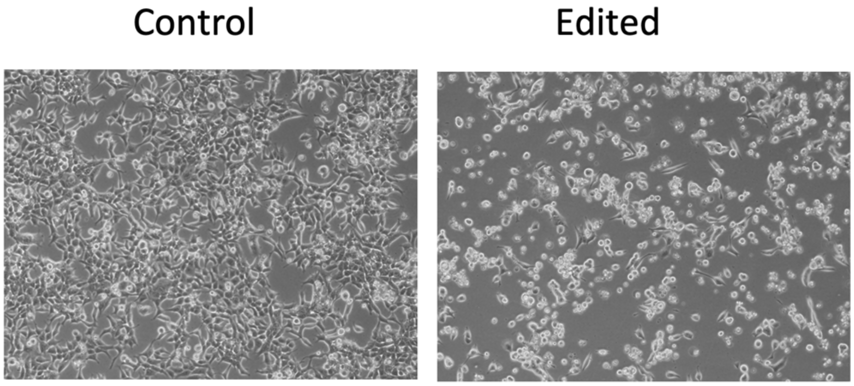

Figure S3: Microscopic images of control and C-terminus truncated PSMA3 edited cells after 6 days.

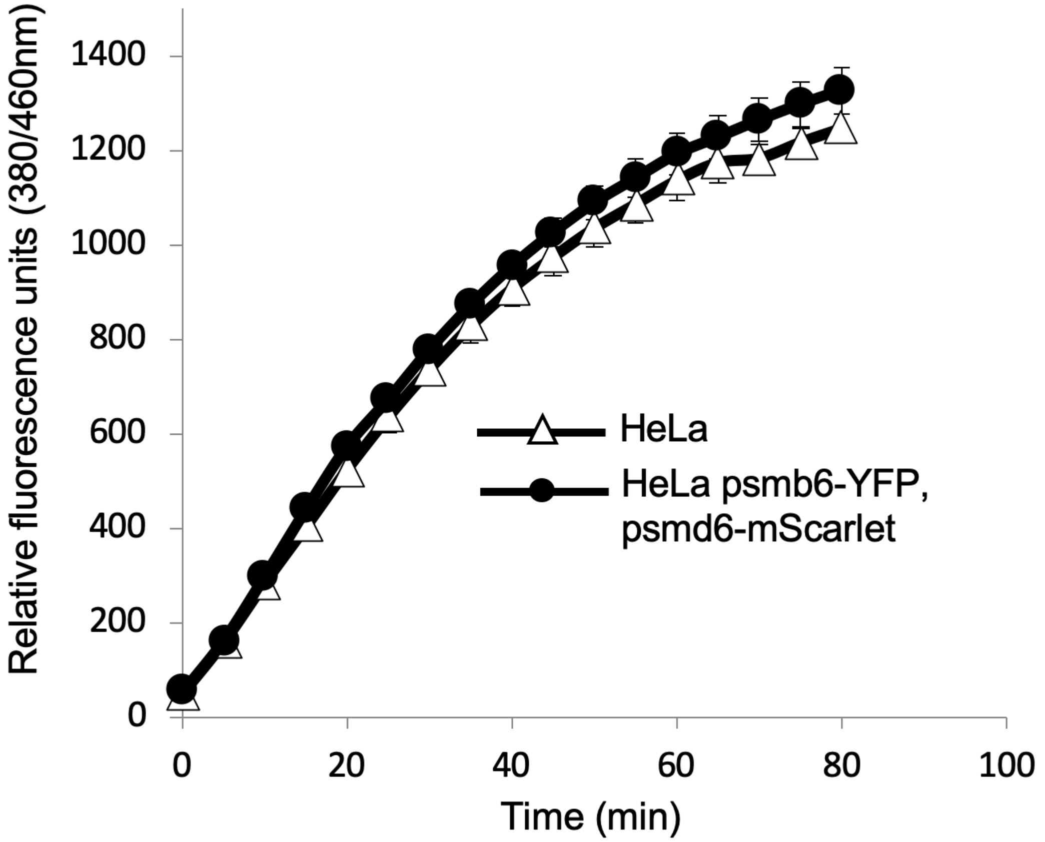

Figure S4: Proteasome activity of HeLa cells edited for PSMB6-YFP and PSMD6-mScarlet. Extracts of control HeLa cells and the CRISPR edited cells were assayed for Chymotrypsin-like activities over time using the fluorescent substrate Suc-LLVY-AMC.

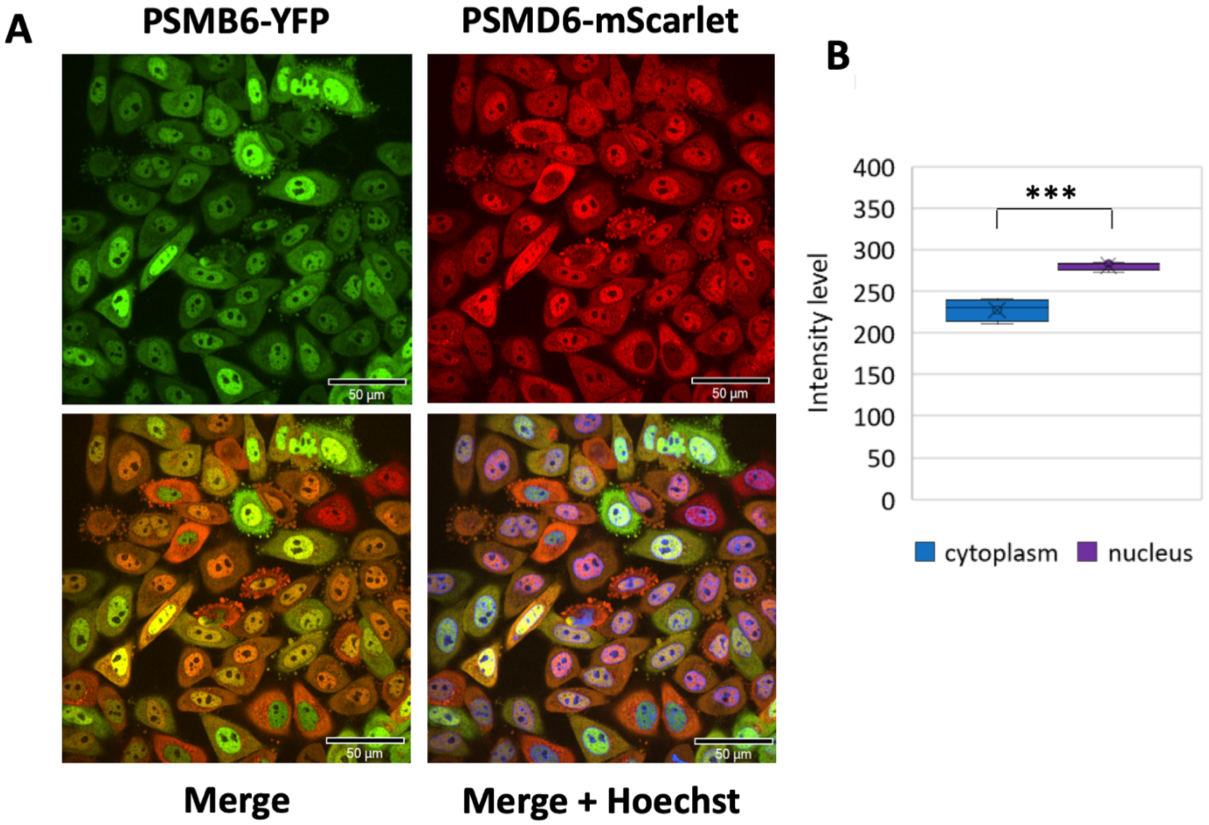

Figure S5: Establishment of a double reporter cell line. A. HeLa PSMB6-YFP PSMB6-mScarlet cells – a wide-field view. Green live-imaging marker represents 20S, red marker 19S and hence 26S, blue marker represents the nucleus. Scale bar is 10µm. B. Boxplot analysis of the intensity level in the cytoplasm and the nucleus. ***P < 0.001 by 2-tailed unpaired t-test.

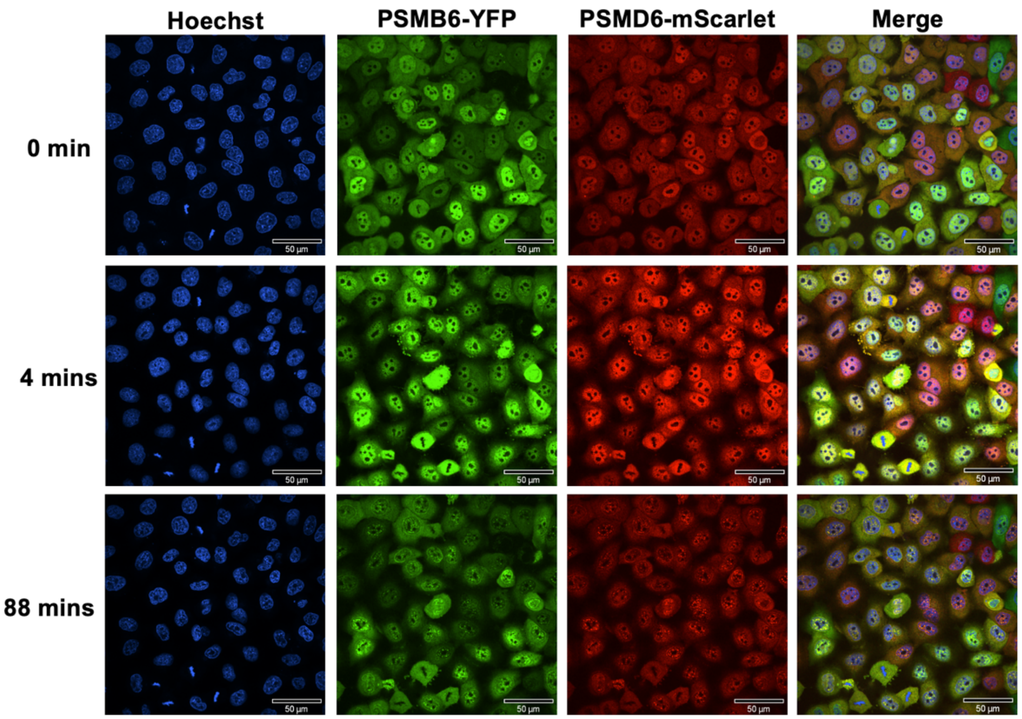

Figure S6: Wide-field images of proteasome granules under sucrose osmotic stress at different time points. Time-lapse images of HeLa PSMB6-YFP PSMD6-mScarlet cells treated with 200 mM sucrose. The green live-imaging marker represents all proteasomes, the red live-imaging marker represents 19S RP and the yellow spots in the merge panel represent 26S proteasomes. The nuclei were Hoechst stained (blue). Selected time points are shown. The scale bar is 10µm.

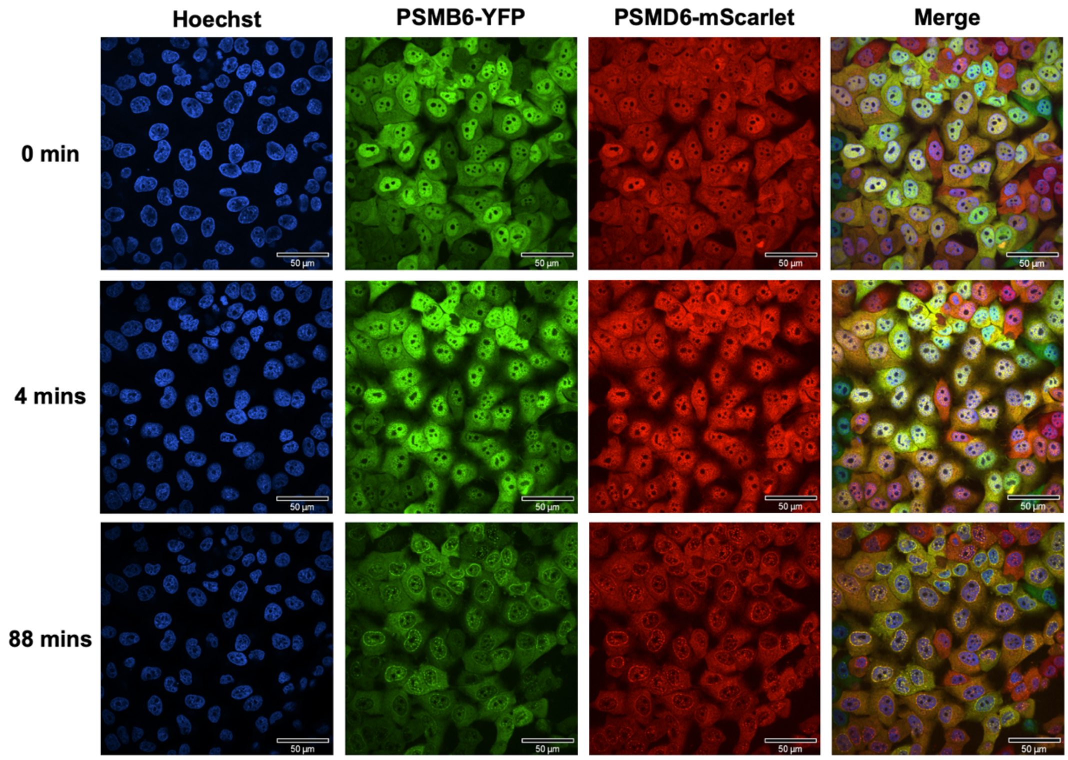

Figure S7: Wide-field images of proteasome granules under NaCl osmotic stress at different time points. Time-lapse images of HeLa PSMB6-YFP PSMD6-mScarlet cells treated with 150 mM NaCl. The green live-imaging marker represents all proteasomes, the red live-imaging marker represents 19S RP and the yellow spots in the merge panel represent 26S proteasomes. The nuclei were Hoechst stained (blue). Selected time points are shown. The scale bar is 10μm.

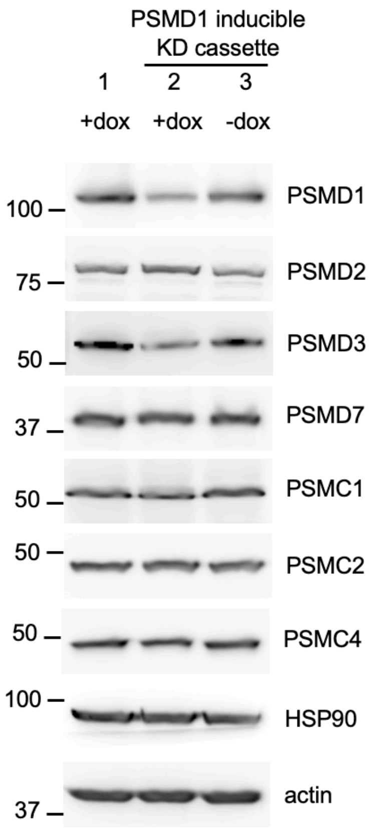

1 HeLa PSMB6-YFP PSMD6-mScarlet + dox control

2 HeLa PSMB6-YFP PSMD6-mScarlet PSMD1-KD cassette + dox

3 HeLa PSMB6-YFP PSMD6-mScarlet PSMD1-KD cassette - dox

Figure S8: Western blot analysis of the components of 19S in PSMD1 knockout cells. HeLa PSMB6-YFP PSMD6-mScarlet PSMD1-KD cassette cells were treated (lane 2) or untreated (lane 3) with doxycycline for 3.5 days. As an additional control, HeLa PSMB6-YFP PSMD6-mScarlet cells without PSMD1-KD cassette were also doxycycline-treated and analyzed after 3.5 days (lane 1).

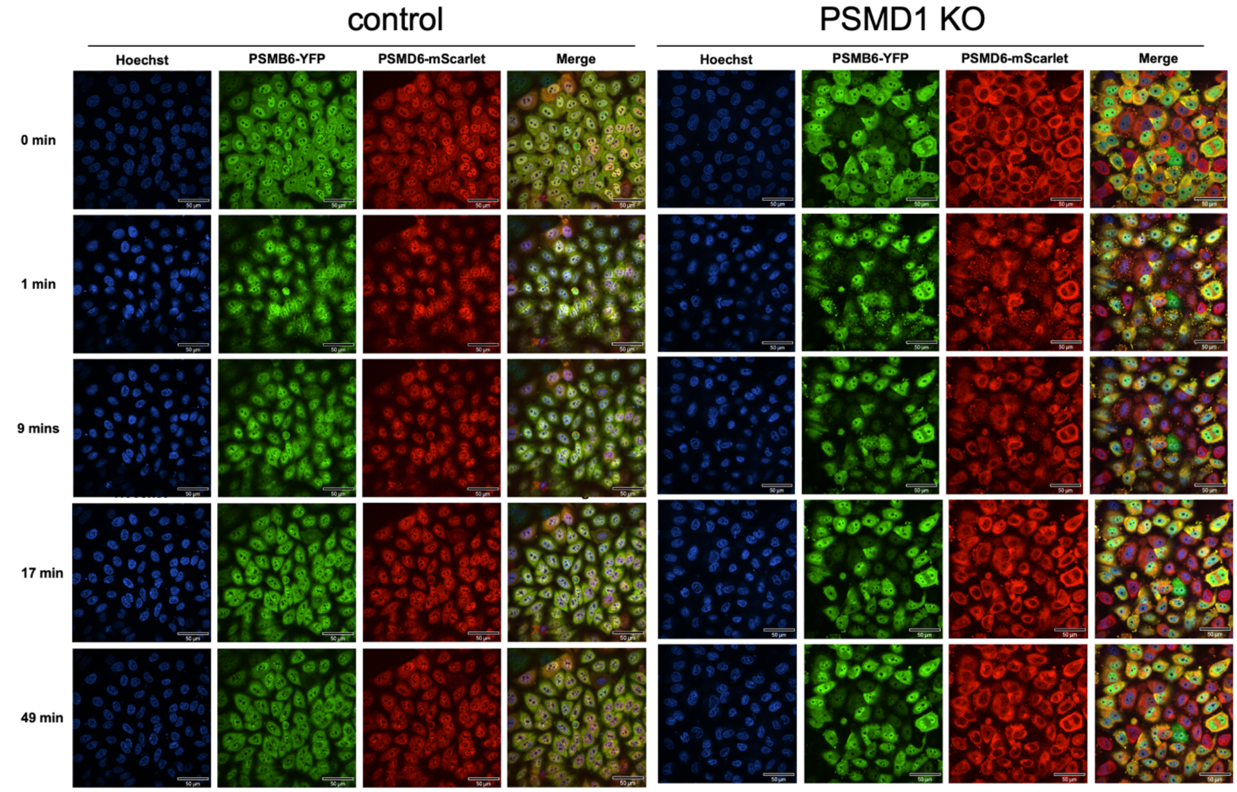

Figure S9: Wide-field images of proteasome granules under osmotic stress. Wide-fields time-lapse images of HeLa PSMB6-YFP PSMD6-mScarlet cells and HeLa PSMB6-YFP PSMD6-mScarlet PSMD1-KD cells treated with 100 mM NaCl. The scale bar is 10µm.
